# Effect of Adenosine and Adenosine Receptor Antagonists on Retinal Müller Cell Inwardly Rectifying Potassium Channels under Exogenous Glutamate Stimulation

**DOI:** 10.1155/2018/2749257

**Published:** 2018-08-29

**Authors:** Zhongjing Lin, Ping Huang, Shouyue Huang, Lei Guo, Xing Xu, Xi Shen, Bing Xie, Yisheng Zhong

**Affiliations:** ^1^Department of Ophthalmology, Ruijin Hospital Affiliated Medical School, Shanghai Jiaotong University, 197 Ruijin Er Road, Shanghai 200025, China; ^2^Shanghai Key Laboratory for Bone and Joint Diseases, Shanghai Institute of Traumatology and Orthopaedics, Ruijin Hospital Affiliated Medical School, Shanghai Jiaotong University, 197 Ruijin Er Road, Shanghai 200025, China

## Abstract

The vitreousness of glaucoma subjects contains elevated glutamate, and excessive extracellular glutamate is toxic to retinal neurons. Therefore, glutamate clearance is potentially impaired in the retina of glaucoma subjects. Müller cells play an important role in maintaining low extracellular levels of neurotransmitters, such as glutamate. A better understanding of the cross-talk between adenosine and glutamate may provide a better characterization of the regulatory network in Müller cells. Here, Müller cells were purified from the rat retina on postnatal day 5 using the papain digestion method. Application of increasing concentrations of glutamate (0-20 mmol/L) caused a dose-dependent decrease in the expression levels of Kir4.1, Kir2.1, GLAST, and GS. Exogenous adenosine regulated Kir channels and subsequently promoted GLAST and GS expression levels in Müller cells under exogenous glutamate stimulation. These effects were partly dependent on adenosine receptors.

## 1. Introduction

Müller cells, the major glial cells in the retina, radially span the entire retina and are closely associated with all retinal cells [1]. In the normal retina, Müller cells are vital in the regulation of synaptic transmission and extracellular levels of neurotransmitters, such as glutamate [2, 3]. Released glutamate in the extracellular space is primarily transported into Müller cells by L-glutamate/L-aspartate transporter (GLAST) and subsequently quickly converted to glutamine by glutamine synthetase (GS). Müller cells are also primarily responsible for buffering K^+^. The inwardly rectifying potassium (Kir) channels expressed on the glial membranes, especially the Kir 4.1 and Kir 2.1 channels, are the most significant transporters mediating K^+^ buffering. Additionally, Kir channels may be involved in glutamate clearance [4, 5].

Increased extracellular concentrations of glutamate can result in abnormal synaptic transmission and are associated with abnormal neural excitability and function in the retina [6–8]. Moreover, increases in glutamate may depolarize Müller cells by a second messenger-linked reduction in K^+^ conductance [9]. When depolarized sufficiently, Müller cells have been shown to reduce glutamate clearance, thus resulting in an increase in the retinal glutamate level. Given elevated glutamate levels in the vitreousness of glaucoma subjects and that excessive levels of extracellular glutamate are toxic to retinal neurons [10–12], glutamate clearance is likely impaired in the retina of glaucoma subjects. Therefore, exploring the agents that could protect Müller cells from the toxic effects of excessive glutamate may provide a better understanding of the regulatory network in Müller cells and shed light on the therapeutic discovery.

Adenosine is an important endogenous regulator in the retina [13, 14]. The actions of adenosine are usually mediated through its interaction with specific receptor subtypes, A_1_, A_2A_, A_2B_, and A_3_ [15]. All these adenosine receptors have been identified in the retina [16, 17] and are also expressed in Müller cells [18]. Adenosine plays a protective role in the pathophysiology of several retinal diseases, such as diabetic retinopathy [19] and glaucoma [20]. These favorable effects have been explored in vitro, and various signaling pathways have been implicated. However, how adenosine influences the expression levels of Kir channels in cultured Müller cells under exogenous glutamate stimulation is relatively unknown.

In the current study, we attempt to investigate whether adenosine can regulate Kir channels and subsequently promote the expression levels of GLAST and GS in Müller cells under exogenous glutamate stimulation.

## 2. Methods

### 2.1. Animals and Ethics Statement

All the experimental procedures were performed in accordance with the guidelines for the Care and Use of Laboratory Animals of the National Institutes of Health. The animal procedures were in accordance with the ARVO Statement for the Use of Animals in Ophthalmic and Vision Research and with approval from the institutional review board of Ruijin Hospital, affiliated with Shanghai Jiao Tong University School of Medicine, Shanghai, China. Five-day-old Sprague-Dawley rats were euthanized by cervical vertebra dislocation. All efforts were made to minimize suffering and the number of rats used in the study.

### 2.2. Culture of Rat Retinal Müller Cells

Pure retinal Müller cell cultures were prepared as previously described with minor modifications [21]. In brief, Müller cells were isolated from retinas collected from 5-day-old Sprague-Dawley rats. The retinas were gently dissociated with 16.5 Units/mL papain (Worthington Biochemical Corp., Lakewood, NJ) and 100 U/mL DNase I (Sigma-Aldrich Corp. St. Louis, MO, USA) for 30 min at 37°C. After removal of the mixed papain solution, the cells were washed and centrifuged in Dulbecco's Minimum Essential Medium (DMEM) (Gibco-BRL, Grand Island, NY, USA) containing 10% fetal bovine serum (FBS). Subsequently, the cells were resuspended in culture medium and plated in 25-cm^2^ culture flasks. Then, the dissociated cells were cultured at 37°C with 5% CO_2_. When approaching 80-100% confluence, the cells were dissociated from the culture flasks using 0.25% trypsin/EDTA (Gibco, Grand Island, NY, USA) and subsequently divided into subcultures. Müller cells were almost purified after 4-6 passages and only primary cells within passage 15 were used for further experiments.

### 2.3. Immunocytochemistry

The identity of Müller cells was confirmed by immunocytochemical analyses using antibodies specific for Müller cells, GS, and glial fibrillary acidic protein (GFAP). Briefly, the cells were fixed with 4% paraformaldehyde for 20 min. Then, the samples were incubated with 0.3% Triton X-100 for 10 min and blocked with 1% bovine serum albumin (BSA) for 30 min. Afterwards, the cells were treated with the following primary antibodies overnight at 4°C: rabbit monoclonal anti-GS (1 : 5000; Abcam) or mouse polyclonal anti-GFAP (1 : 200, Abcam). On the following day, the samples were further incubated with the following secondary antibodies: Cy3-labeled donkey anti-rabbit IgG (1 : 200, Biolegend) or FITC-labeled donkey anti-mouse IgG (1 : 200, Jackson ImmunoResearch, PA, USA), for 2 hours at room temperature. Then, the cells were washed again three times for 10 min each with PBS and stained with 1 *μ*g/mL DAPI (1 : 100, Sigma-Aldrich, St. Louis, MO, USA) for 10 min at room temperature. Afterwards, the immunoreactive fluorescence staining of the cells was photographed using a Zeiss Imager M1 laser scanning microscope (Carl Zeiss, Germany).

### 2.4. Materials

DPCPX (8-cyclopen-tyl-1,3-dipropylxanthine), ZM241385 (4-(2-[7-amino-2-{2-furyl}{1,2,4}triazolo{2,3-a}{1,3,5}triazin-5-ylamino]ethyl)phenol), MRS1191 (3-ethyl-5-benzyl-2-methyl-4-phenylethynyl-6-phenyl-1,4-(±)-dihydropyridine-3,5-dicarboxylate), and SCH442416 (5-amino-2-(2-furyl)-7-[3-(4-methoxyphenyl)propyl]-7H-pyrazolo[4,3-e][1,2,4]triazolo[1,5-c]pyrimidine) were purchased from Sigma-Aldrich. All the compounds were prepared at a concentration of 10 mmol/L according to the manufacturer's instructions, and then the stock solutions were stored at -20°C.

### 2.5. Western Blotting

Cell samples were homogenized in radio-immunoprecipitation assay (RIPA) buffer supplemented with 1 mM phenylmethylsulfonyl fluoride (PMSF). After the samples were centrifuged at 12000 g for 15 min at 4°C, the protein concentrations of the supernatants were quantified with a BCA Protein Assay Kit (Pierce Biotechnology, Rockford, IL). All the protein samples were boiled at 100°C for 10 min and then stored at -80°C. Aliquots of total protein samples (30 *μ*g) were separated using 10% sodium dodecyl sulfate-polyacrylamide gel electrophoresis (SDS-PAGE) and then transferred onto polyvinyldifluoride membranes (PVDF; Merck Millipore, Darmstadt, Germany). After the membranes were blocked in 5% BSA for 1 hour at room temperature, they were treated with the following primary antibodies overnight at 4°C: rabbit polyclonal anti-Kir4.1 (1 : 300 dilution; Abcam), rabbit monoclonal anti-Kir2.1 (1 : 1000 dilution; Abcam), rabbit polyclonal anti-GLAST (1 : 200 dilution; Abcam), rabbit monoclonal anti-GS (1 : 10000 dilution; Abcam), and mouse monoclonal anti-GAPDH (1 : 10000 dilution, Kangchen). On the following day, the membranes were incubated with the following secondary antibodies for 90 min at room temperature: HRP-conjugated goat anti-rabbit IgG (1 : 5000; Abcam) or anti-rat IgG (1 : 2000; Abcam). Subsequently, the protein bands were revealed by enhanced chemiluminescence (ECL) using an ImageQuant Las 4000 mini system.

### 2.6. Real Time-PCR

To examine the mRNA expression levels of Kir4.1, Kir2.1, GLAST, and GS in Müller cells after various treatments, we performed real time-polymerase chain reaction (RT-PCR). Briefly, total RNA was collected from the cell samples using TRIzol reagent (Invitrogen, Carlsbad, CA, USA) in accordance with the manufacturer's protocol. Complementary DNA was prepared by reverse transcription from mRNA using a PrimeScript RT Master Mix Kit (Takara Bio, Inc., Shiga, Japan). Subsequently, RT-PCR was performed using SYBR® Premix Ex Taq™ II (Tli RNaseH Plus; Takara, Japan) according to the following thermal cycling conditions: the initial denaturation with one cycle at 95°C for 30 s; followed by amplification with 40 cycles at 95°C for 5 s and 60°C for 30 s; and the melting curve stage with temperatures ranging from 60°C to 95°C. RT-PCR was performed using the following primers (5′ to 3′): GAPDH (F): ATGACTCTACCCACGGCAAG, (R): TACTCAGCACCAGCATCACC, Kir4.1 (F): AAAGAAGAGGGCTGAGACGA, (R): AAGCAGTTTGCCTGTCACCT, Kir2.1 (F): CGTGGGAGAGAAAGGACAGA, (R): CCAAAGAACAGCCAGGAGAG, GLAST (F): CCTATGTGGCAGTCGTTT, (R) CTGTGATGGGCTGGCTAA, GS (F): CCGCTCTTCGTCTCGTTC, (R): CTGCTTGATGCCTTTGTT. The double delta C_T_ analysis was used when we analyzed the RT-PCR data.

### 2.7. Statistical Analysis

Statistical analyses were conducted using GraphPad Prism 6.0 (GraphPad Software, Inc., San Diego, CA, USA). One-way analyses of variance (ANOVA) were performed to compare the data of the various groups followed by Bonferroni post hoc test. P < 0.05 indicates statistically significant difference for all the comparisons. The error bars in the figures indicate the standard error of the mean (SEM). The term “n” refers to the number of replicates.

## 3. Results

### 3.1. Isolation and Primary Culture of Müller Cells

**Figure 1:**
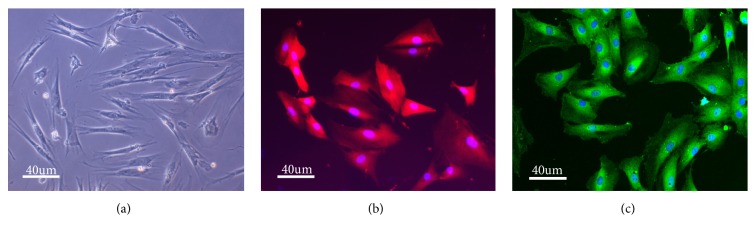
The identification of rat retinal Müller cells. (a) The morphology of rat retinal Müller cells observed using a light microscope. (b) Rat retinal Müller cells identified by GS immunocytochemical staining (red). (c) Rat retinal Müller cells identified by GFAP immunocytochemical staining (green).

Repeated media changes and cell passaging successfully removed nonadherent cells and retinal debris. Müller cells were almost purified after 4-6 passages in cell cultures. Cultured Müller cells were mostly spindle-shaped or polygonal and exhibited centrally located nuclei (Figure 1(a)). GS (Figure 1(b)) and GFAP (Figure 1(c)) immunocytochemical labeling indicated that the cultured cells were Müller cells and confirmed the high purity of Müller cell cultures.

### 3.2. The Effect of Glutamate and Adenosine on Müller Cells

**Figure 2:**
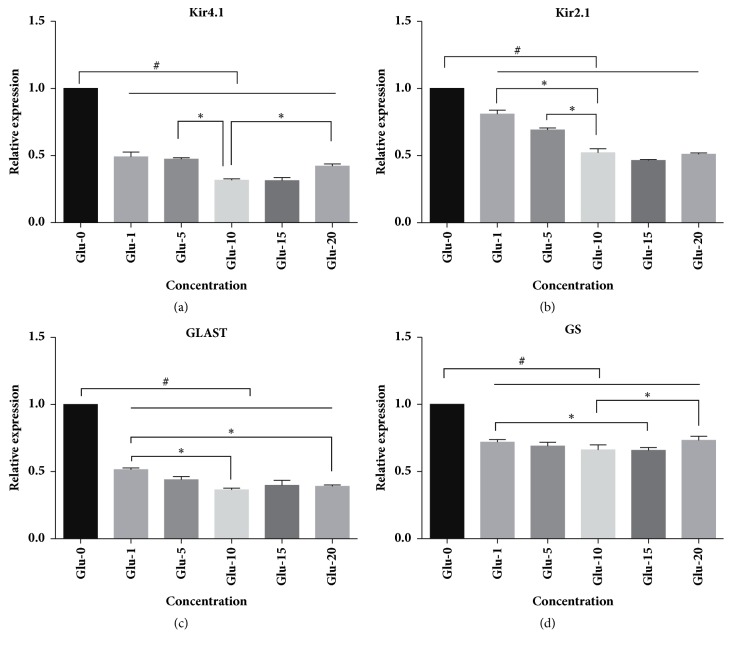
The effect of glutamate treatment (Glu, mmol/L) on cultured rat Müller cells as determined by real time-PCR. The cultures were treated with different concentrations of glutamate (0-20 mmol/L) when approaching 80-100% confluence. Application of increasing concentrations of glutamate caused a dose-dependent decrease in the mRNA expression levels of Kir4.1, Kir2.1, GLAST, and GS. The effects were most remarkable when glutamate concentration was increased to 10 mmol/L (n=3, all P<0.05). *∗* indicated P<0.05 between the two groups. # indicated P<0.05 between the blank control group and any of the glutamate treatment groups.

**Figure 3:**
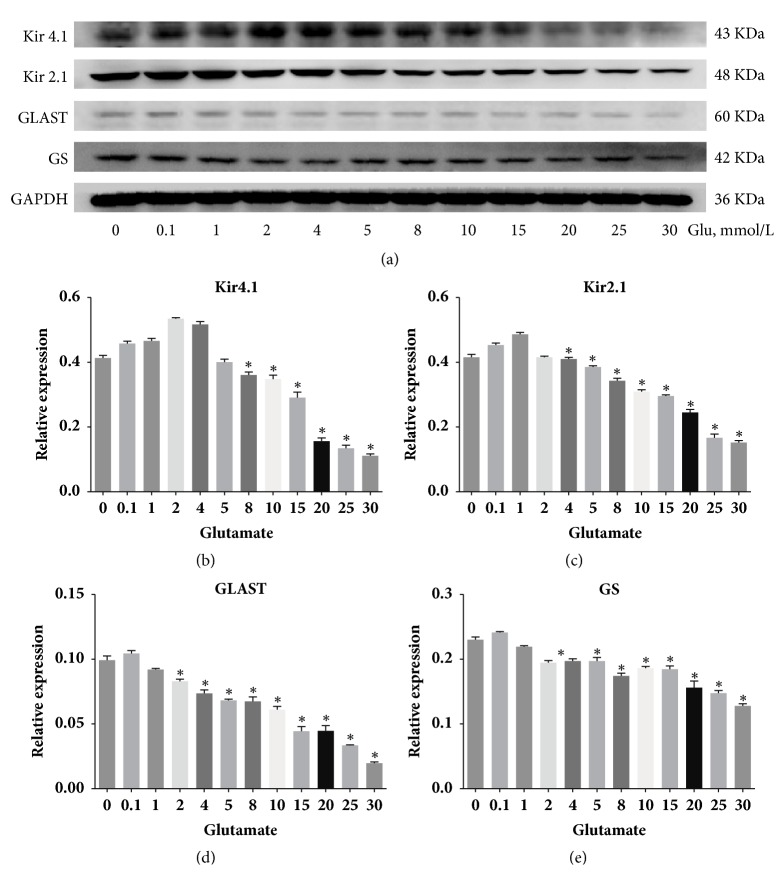
The effect of glutamate treatment (Glu, mmol/L) on cultured rat Müller cells as determined by western blotting analysis. The results indicated a concentration-dependent effect of glutamate in the protein expression levels of Kir4.1, Kir2.1, GLAST, and GS. *∗* indicated P<0.05 compared with the blank control group using independent sample t-test (n=3).

**Figure 4:**
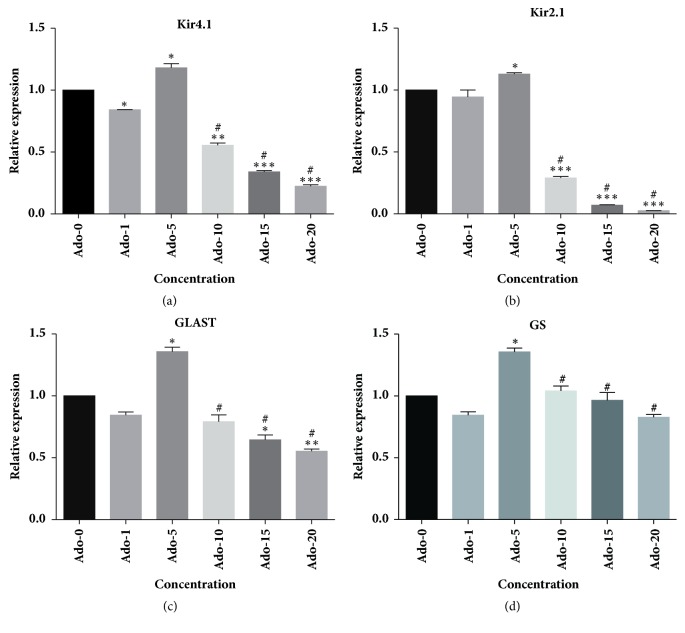
The effect of adenosine (Ado, *μ*mol/L) on responses to 10 mmol/L-glutamate in cultured retinal rat Müller cells as determined by real time-PCR. The cultures received combined treatment of 10 mmol/L-glutamate and different concentrations of adenosine. The mRNA expression levels of Kir4.1, Kir2.1, GLAST, and GS were significantly increased by the addition of 5 *μ*mol/L adenosine (n=3, all P<0.05). Continuing increase in the concentration caused decreases in mRNA expression levels. *∗* indicated P<0.05 compared with the Ado-0 group. *∗∗* indicated P<0.01 compared with the Ado-0 group. *∗∗∗* indicated P<0.01 compared with the Ado-0 group. # indicated significant difference compared with the Ado-5 group.

To determine the effect of glutamate on Müller cells, we performed mRNA expression profiling after a 24-hour treatment with medium or different concentrations of glutamate (0-20 mM) (Figure 2). RT-PCR analysis demonstrated that compared with medium control, glutamate stimulation caused a significant reduction in the expression levels of Kir4.1, Kir2.1, GLAST and GS mRNA (n=3, all P<0.05). The effects were most remarkable when the glutamate concentration was increased to 10 mmol/L. In addition, a wider range of glutamate concentrations was further used in the western blotting analysis (Figure 3). The results further confirmed a concentration-dependent effect of glutamate. Subsequently, different concentrations of adenosine were applied to cultured Müller cells in the combination of 10 mmol/L-glutamate for 24 hours (Figure 4). When we compared results from the glutamate group with those from the glutamate plus adenosine treatment groups, we found that Kir4.1, Kir2.1, GLAST, and GS mRNA expression levels were increased by the addition of 5 *μ*mol/L adenosine (n=3, all P<0.05). Continuing increase in the concentrations would cause decreases in the mRNA expression levels, indicating that adenosine modulated glutamate-induced mRNA expression in a concentration-dependent manner. This concentration (5 *μ*mol/L adenosine) was selected for further experiments.

### 3.3. The Effect of Adenosine and Adenosine Receptor Antagonists on Cultured Müller Cells under Exogenous Glutamate Stimulation

To further elucidate the role of adenosine receptors, Müller cells were incubated for 24 hours by combined treatment with glutamate stimulation (10 mmol/L) and 5 *μ*mol/L adenosine or 5 *μ*mol/L adenosine plus 10 *μ*mol/L of a selective adenosine receptor antagonists, while the controls were cultured with only glutamate (10 mmol/L) in the absence of adenosine or adenosine receptor antagonists. The specific subtypes of adenosine receptor antagonists used in our study included DPCPX (A_1_), ZM241385 (A_2A_), MRS1191 (A_3_), and SCH442416 (A_2A_).

**Figure 5:**
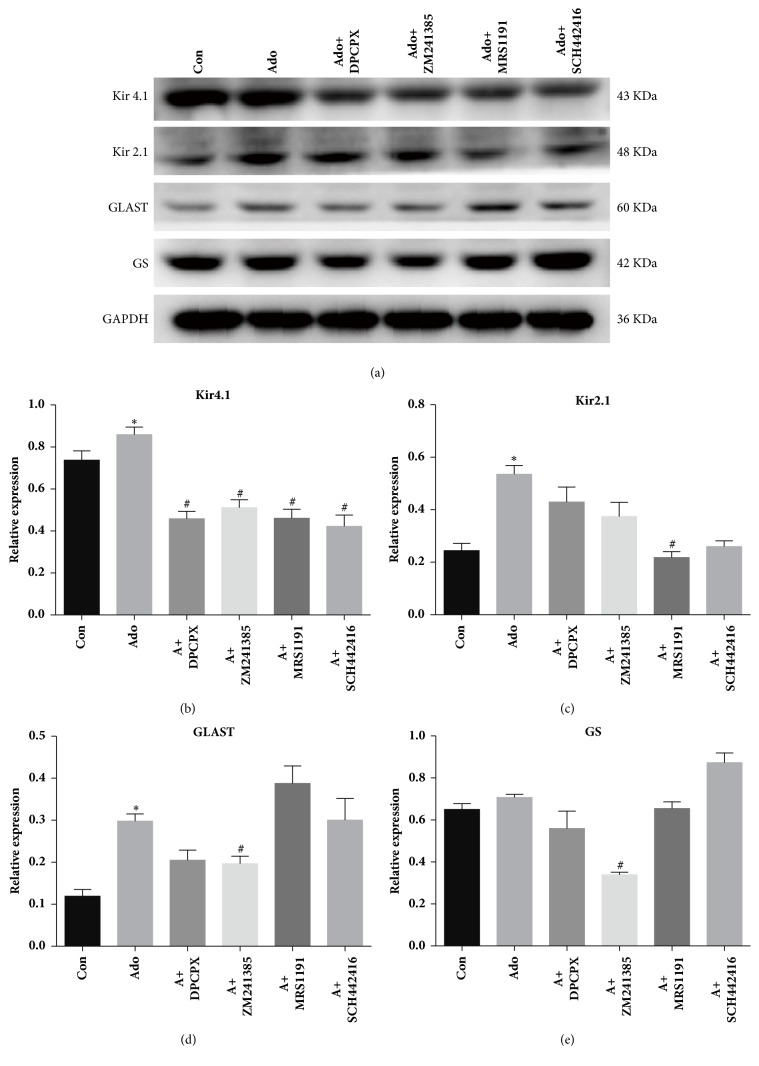
The effect of adenosine and adenosine receptor antagonists on cultured Müller cells under exogenous glutamate stimulation as determined by western blotting analysis. The cultures were treated with the combination of glutamate and adenosine or adenosine plus any of adenosine receptor antagonists, while the controls were cultured only with glutamate. *∗* indicated P<0.05 compared with the control group. # indicated P<0.05 compared with the Ado group.

**Figure 6:**
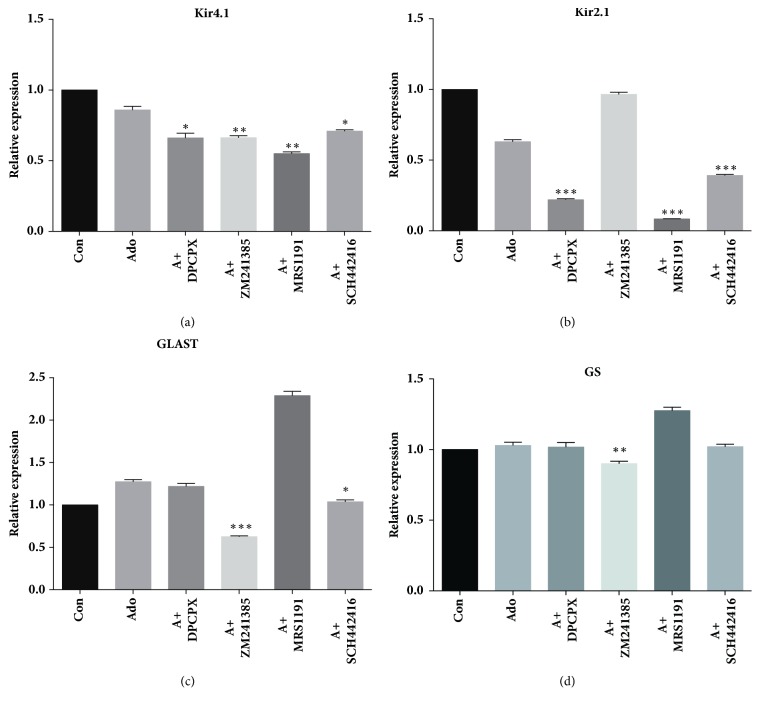
The effect of adenosine and adenosine receptor antagonists on cultured Müller cells under exogenous glutamate stimulation as determined by RT-PCR analysis. The cultures were treated with the combination of glutamate and adenosine or adenosine plus any of adenosine receptor antagonists, while the controls were cultured only with glutamate. *∗* indicated P<0.05 compared with the Ado group, *∗∗* indicated P<0.01 compared with Ado group, and *∗∗∗* indicated P<0.001 compared with the Ado group.

By 24 hours in culture, compared with the adenosine group, the DPCPX, ZM241385, MRS1191, and SCH442416 groups exhibited significant reductions in Kir4.1 protein expression levels by 46.65%, 40.54%, 46.33%, and 50.85%, respectively (n=3, p=0.0195, p=0.0118, p=0.0305, and p=0.0354, respectively) (Figure 5(b)). The mRNA expression levels were downregulated accordingly by 20.38%, 20.37%, 30.92%, and 22.14%, respectively (n=6, p=0.0367, p=0.0023, p=0.0010, and p=0.0305, respectively) (Figure 6(a)). With regard to the changes in Kir2.1, only the protein levels in the MRS1191 group were significantly decreased (n=3, p=0.0427), while no significant changes were observed in the other three adenosine antagonist groups (Figure 5(c)). However, Kir2.1 mRNA expression levels in the DPCPX, MRS1191, and SCH442416 groups were downregulated by 65.16%, 86.72%, and 37.91% (n=6, all p<0.001) (Figure 6(b)). Overall, these data indicated that all these adenosine receptor antagonists could block the effect of adenosine on Kir4.1 expression, whereas only the A_3_ receptor antagonist MRS1191 could block the effect of adenosine on Kir2.1 expression.

By 24 hours in culture, compared with the adenosine group, the ZM241385 group showed a 33.98% decrease in GLAST protein levels (n=3, P=0.0021) (Figure 5(d)), whereas the ZM241385 and SCH442416 groups exhibited 50.95% and 18.61% decreases, respectively, in mRNA expression levels (n=6, p<0.0001, and p=0.402, respectively) (Figure 6(c)). Similarly, the GS protein and mRNA expression levels in the ZM241385 group were significantly decreased by 52.10% and 12.48%, respectively (n=3, P=0.0399; n=6, P=0.0033, respectively) (Figures 5(e) and 6(d)). No significant changes were observed in any other adenosine antagonist group. These results strongly suggested that the effects of adenosine on GLAST and GS expression levels mainly occur via the adenosine A_2A_ receptor.

## 4. Discussion

Müller glial cells are of vital importance to maintain the retinal physiological environment, including the regulation of extracellular glutamate and K^+^ levels. An established method was employed to obtain primary Müller cell cultures. Müller glial cells were acutely isolated from the retina of rats at 5 postnatal days. Previous studies have indicated that Müller glial cells do not express Kir channels and glutamate transporters until 11-28 postnatal days [22, 23]. However, we used much older cells than 5th day after the birth because the cells were differentiating in culture. Further important phenotypic characteristics of differentiated Müller cells are developed, such as Kir channels and glutamate transporters [24, 25].

The presence of glutamate caused a significant and dose-dependent reduction in Kir channels, GLAST, and GS expression levels in cultured Müller cells, consistent with previous studies demonstrating glutamate-induced toxicity in other primary cell cultures [26, 27]. However, this decline is not due to cell death since cultured Müller cells can withstand glutamate treatment from 0.1 to 20 mM concentrations for 48 hours [26]. Müller cells are essential for maintaining the retinal microenvironment and can respond to abnormally elevated glutamate levels, but when extracellular glutamate reaches toxic levels, Müller cells may be unable to efficiently take up excess extracellular glutamate, conversely, with a return of malfunction.

Glutamate is a neurotransmitter which acts at a range of ionotropic and metabotropic glutamate receptors to exert its physiological roles. The ionotropic glutamate receptors include N-methyl-D-aspartate (NMDA) receptors, a-amino-3-hydroxy-5-methyl-4-isoaxazolepropionate (AMPA) receptors, and kainate receptors [28]. It has become evident that, in addition to glutamate receptors, the uptake of glutamate by glia cells via specific transporters is also a key component of synaptic transmission and excitotoxic processes, maintaining a physiological extracellular glutamate level and thus regulating the balance between signaling and pathology. GLAST, the predominant transporter, is localized in Müller cells in the retina. Previous study suggested that glutamate receptors activation was involved in GLAST regulation in a human-derived Müller cell line [29]. Group I metabotropic glutamate receptor agonist modulated Kir4.1 protein and mRNA in Müller cells [30, 31]. In addition, activation of NMDA receptor-channels in human retinal Müller glial cells inhibits inward-rectifying potassium currents. The noncompetitive NMDA antagonist, MK-801, could block the effect, while there was no significant block by CNQX, an AMPA/kainate antagonist [32]. All these results supported that the effect of glutamate may encompass signaling induced by receptor activation.

In our in vitro study, adenosine, if proper concentration was administered, could improve the expression levels of Kir channels, GLAST and GS in Müller cells under exogenous glutamate stimulation. Meanwhile, different concentrations of adenosine had complicated actions, and concentrations higher than 5 *μ*M significantly prevented these effects, suggesting adenosine would be effective at a selected concentration range, which was correlated with the experimental conditions. Actually, when cells are exposed to toxic substance such as glutamate, intracellular ATP released from cell interior will be rapidly and sequentially degraded to ADP and this to AMP which in turn is dephosphorylated by 5′-nucleotidase to adenosine; consequently, an amount of adenosine may be formed, triggering its actions [18, 33]. Moreover, adenosine can be metabolized in the extracellular medium by adenosine deaminase to form inosine. Previous work has observed a significant rescue of neurons in cultures exposed to glutamate when pretreated with adenosine plus EHNA, an inhibitor of adenosine deaminase, indicating adenosine itself could protect retinal neurons against glutamate excitotoxicity [34]. In a word, in addition to glutamate recycling, adenosine recycling in Müller cells might occur. The cross-talk between adenosine and glutamate is complicated.

Adenosine is one of the most prominent endogenous mediators, and the current consensus is that the regulatory effects of adenosine are predominantly mediated by its receptors [35]. Therefore, we first examined whether adenosine exerted these regulatory influences through its receptors. The effects of adenosine on Kir4.1 and Kir2.1 expression levels were partly inhibited by different receptor antagonists, suggesting that these changes were not specific to adenosine receptor subtypes. However, we speculated that the effects of adenosine on GLAST and GS expression levels were mainly inhibited by the A_2A_ receptor antagonist, while the A_1_ receptor and A_3_ receptor antagonists were not involved. Moreover, the effects were strongly antagonized by ZM241385 but much less so by SCH442416 at a concentration that is expected to be selective for A_2A_ receptors, suggesting that A_2A_ receptor antagonists, commonly thought to be pharmacologically similar, may act differently. When trying to explain the differential actions of A_2A_ receptor antagonists observed in vitro, it is interesting and worthwhile to note that ZM241385 is also known to be an inverse agonist and displayed moderate affinity for the A_2B_ receptor [36, 37], which might contribute to the alterations. Moreover, the decreased protein expression levels were not entirely consistent with a corresponding reduction in mRNA levels, indicating that the suppressed expression levels of these proteins are, at least partially, disturbed at the transcription level. Besides, posttranscriptional regulation may have a role to play from mRNA level to protein level. After translation into protein, there are also posttranslational modifications and regulations, all of which form a complex regulatory network.

In the retina, Müller cells are highly responsible for removing the bulk of glutamate from the extracellular space. Impaired glutamate recycling in Müller cells is considered a common etiological factor in retinopathies and is associated with excessive loads of glutamate in the retina. Previous studies have demonstrated that adenosine has favorable effects on various retinal cells [38–42]. In our current study, adenosine may act as a protective agent by modulating glutamate uptake, as Kir channels and glutamate transporters were markedly increased. The upregulation of Kir channels could accelerate K^+^ clearance to prevent the excessive release of glutamate. The increased expression levels of GLAST and GS could induce a rapid upregulation of glutamate uptake. However, our study has some limitations. We did not thoroughly investigate the modulation of K^+^ currents in response to adenosine or the intracellular mechanisms underlying the influence of adenosine receptor activation on Kir channels due to the difficulty. In addition, the cellular signal transduction pathways involved in the actions of adenosine remain to be elucidated. Although prior experimental work has been dedicated to elucidating the signaling mechanism following A_2A_ receptor activation [43, 44], the signal transduction pathway remains largely enigmatic. Further studies are warranted to delineate the intracellular signaling pathways leading to the regulatory effects of adenosine on Müller cells under exogenous glutamate stimulation, as well as determine whether adenosine exerts those regulatory effects in an in vivo glaucoma models.

## 5. Conclusion

Exogenous adenosine regulated Kir channels and subsequently promoted GLAST and GS expression levels in Müller cells under the exogenous glutamate stimulation. These effects were partly dependent on adenosine receptors.

## Data Availability

All relevant data are within the paper.
